# Cryptic species of *Curvularia* in the culture collection of the Queensland Plant Pathology Herbarium

**DOI:** 10.3897/mycokeys.35.25665

**Published:** 2018-06-15

**Authors:** Yu Pei Tan, Pedro W. Crous, Roger G. Shivas

**Affiliations:** 1 Biosecurity Queensland, Department of Agriculture and Fisheries, Ecosciences Precinct, Dutton Park, QLD 4102, Australia; 2 Westerdijk Fungal Biodiversity Institute, Uppsalalaan 8, 3584 CT, Utrecht, Netherlands; 3 Microbiology, Department of Biology, Utrecht University, Padualaan 8, 3584 CT Utrecht, Netherlands; 4 Centre for Crop Health, University of Southern Queensland, Toowoomba, QLD 4350, Australia

**Keywords:** Dothideomycetes, multigene phylogeny, taxonomy, 13 new species

## Abstract

Several unidentified specimens of *Curvularia* deposited in the Queensland Plant Pathology Herbarium were re-examined. Phylogenetic analyses based on sequence data of the internal transcribed spacer region, partial fragments of the glyceraldehyde-3-phosphate dehydrogenase and the translation elongation factor 1-α genes, supported the introduction of 13 novel *Curvularia* species. Eight of the species described, namely, *C. beasleyi* **sp. nov.**, *C. beerburrumensis* **sp. nov.**, *C. eragrosticola* **sp. nov.**, *C. kenpeggii* **sp. nov.**, *C. mebaldsii* **sp. nov.**, *C. petersonii* **sp. nov.**, *C. platzii* **sp. nov.** and *C. warraberensis* **sp. nov.**, were isolated from grasses (Poaceae) exotic to Australia. Only two species, *C. lamingtonensis* **sp. nov.** and *C. sporobolicola* **sp. nov.**, were described from native Australian grasses. Two species were described from hosts in other families, namely, *C. coatesiae* **sp. nov.** from *Litchi chinensis* (Sapindaceae) and *C. colbranii* **sp. nov.** from *Crinum zeylanicum* (Amaryllidaceae). *Curvularia reesii* **sp. nov.** was described from an isolate obtained from an air sample. Furthermore, DNA sequences from ex-type cultures supported the generic placement of *C. neoindica* and the transfer of *Drechslera boeremae* to *Curvularia*.

## Introduction

*Curvularia* is a species-rich genus of pathogens and saprobes associated with plant, human and animals worldwide (Sivanesan 1987, Hyde et al. 2014, Madrid et al. 2014, Manamgoda et al. 2015, Marin-Felix et al. 2017a, b). *Curvularia* species have also been reported from substrates such as air (Almaguer et al. 2012, Hargreaves et al. 2013), aquatic environments (Verma et al. 2013, Su et al. 2015, Sharma et al. 2016) and soil (Manamgoda et al. 2011, Marin-Felix et al. 2017a).

Species delimitation within *Curvularia* based solely on morphology is difficult as many species share similar characters and have overlapping conidial dimensions. Currently, there are 131 species of *Curvularia* (excluding varieties) listed in *Index Fungorum* (accessed on 4 January 2018). Phylogenetic studies based on multilocus sequence analyses of ex-type or reference cultures have recently delimited many cryptic species (Deng et al. 2014, Manamgoda et al. 2014, Tan et al. 2014, Manamgoda et al. 2015, Marin-Felix et al. 2017a, 2017b). Presently, there are 81 accepted species for which taxonomic placement has been established by DNA barcodes to allow accurate identification and comparison (Marin-Felix et al. 2017a, b).

In Australia, 64 species of *Curvularia* have been reported (DAF Biological Collections 2018, Farr and Rossman 2018). Of these, 17 species were described from Australia, namely *C. australiensis*, *C. australis*, *C. bothriochloae*, *C. crustacea*, *C. dactyloctenii*, *C. graminicola*, *C. harveyi*, *C. heteropogonis*, *C. micrairae*, *C. ovariicola*, *C. perotidis*, *C. queenslandica*, *C. ravenelii*, *C. richardiae*, *C. ryleyi*, *C. sorghina* and *C. tripogonis*. Eight of the Australian *Curvularia* species were originally placed in the closely related genus, *Bipolaris*, before transfer to *Curvularia* based on molecular studies (Manamgoda et al. 2012, 2014, Tan et al. 2014).

In this study, 17 unidentified isolates of *Curvularia* maintained in the culture collection held in the Queensland Plant Pathology Herbarium (BRIP) were compared with ex-type and reference isolates. Thirteen new species of *Curvularia* were revealed based on multilocus phylogenetic analyses and are formally described here. In addition, phylogenetic analyses of ex-type cultures have confirmed the placement of a *Curvularia* species, as well as the introduction of a new combination.

## Materials and methods

### Isolates and morphology

Unidentified isolates of *Curvularia* were obtained from BRIP (Table 1), which retains cultures in a metabolically inactive state at -80 °C in a sterile solution of 15% v/v glycerol. In order to observe conidia and conidiophores, living cultures were grown on sterilised leaf pieces of *Zea mays* on modified Sachs agar and on sterilised wheat straws on water agar, incubated at room temperature (approx. 25 °C) for seven days and exposed to near ultraviolet light on a 12 h light/dark diurnal cycle (Sivanesan 1987). Conidia and conidiophores were mounted on glass slides in lactic acid (100% v/v). Images were captured with a Leica DFC 500 camera attached to a Leica DM5500B compound microscope with Nomarski differential interference contrast illumination. Conidial widths were measured at the widest part of each conidium. Means and standard deviations (SD) were calculated from at least 20 measurements. Ranges were expressed as (minimum value–) mean-SD – mean+SD (−maximum value) with values rounded to 0.5 μm.

**Table 1.** *Curvularia* isolates examined.

*Species*	Isolate no.^1^	*Host*	Location	GenBank accession numbers^2^		
				ITS	*gapdh*	*tef1a*
*Bipolaris maydis*	CBS 136.29 ^T^	*Zea mays*	USA	AF071325	KM034846	KM093794
*Curvularia aeria*	CBS 294.61 ^T^	air	Brazil	HF934910	HG779148	–
*C. affinis*	CBS 154.34 ^T^	unknown	Indonesia	KJ909780	KM230401	KM196566
*C. akaii*	CBS 317.86	unknown	Japan	KJ909782	KM230402	KM196569
*C. akaiiensis*	BRIP 16080 ^T^	unknown	India	KJ415539	KJ415407	KJ415453
*C. alcornii*	MFLUCC 10-0703 ^T^	*Zea mays*	Thailand	JX256420	JX276433	JX266589
*C. americana*	UTHSC 08-3414 ^T^	*Homo sapiens*	USA	HE861833	HF565488	–
*C. asiatica*	MFLUCC 10-0711 ^T^	*Panicum* sp.	Thailand	JX256424	JX276436	JX266593
*C. australiensis*	BRIP 12044 ^T^	*Oryza sativa*	Australia	KJ415540	KJ415406	KJ415452
*C. australis*	BRIP 12521 ^T^	*Sporobolus caroli*	Australia	KJ415541	KJ415405	KJ415451
*C. bannonii*	BRIP 16732 ^T^	*Jacquemontia tamnifolia*	USA	KJ415542	KJ415404	KJ415450
***C. beasleyi* sp. nov.**	BRIP 10972 ^T^	*Chloris gayana*	Australia	**MH414892**	**MH433638**	**MH433654**
	BRIP 15854	*Leersia hexandra*	Australia	**MH414893**	**MH433639**	**MH433655**
***C. beerburrumensis* sp. nov.**	BRIP 12942 ^T^	*Eragrostis bahiensis*	Australia	**MH414894**	**MH433634**	**MH433657**
	BRIP 12555	*Eragrostis sororia*	Australia	**MH414895**	**MH433640**	**MH433656**
***C. boeremae* comb. nov.**	IMI 164633 ^T^	*Portulaca oleracea*	India	**MH414911**	**MH433641**	–
*C. borreriae*	MFLUCC 11-0422	unknown Poaceae	Thailand	KP400638	KP419987	KM196571
*C. bothriochloae*	BRIP 12522 ^T^	*Bothriochloa bladhii*	Australia	KJ415543	KJ415403	KJ415449
*C. brachyspora*	CBS 186.50	Soil	India	KJ922372	KM061784	KM230405
*C. buchloës*	CBS 246.49 ^T^	*Buchloë dactyloides*	USA	KJ909765	KM061789	KM196588
*C. carica-papayae*	CBS 135941 ^T^	*Carica papaya*	India	HG778984	HG779146	–
*C. chiangmaiensis*	CPC 28829 ^T^	*Zea mays*	Thailand	MF490814	MF490836	MF490857
*C. chlamydospora*	UTHSC 07-2764 ^T^	*Homo sapiens*	USA	HG779021	HG779151	–
***C. coatesiae* sp. nov.**	BRIP 24170	air	Australia	**MH414896**	**MH433635**	**MH433658**
	BRIP 24261 ^T^	*Litchi chinensis*	Australia	**MH414897**	**MH433636**	**MH433659**
*C. clavata*	BRIP 61680b	*Oryza rufipogon*	Australia	KU552205	KU552167	KU552159
*C. coicis*	CBS 192.29 ^T^	*Coix lacryma-jobi*	Japan	AF081447	AF081410	JN601006
***C. colbranii* sp. nov.**	BRIP 13066 ^T^	*Crinum zeylanicum*	Australia	**MH414898**	MH433642	MH433660
*C. crustacea*	BRIP 13524 ^T^	*Sporobolus* sp.	Indonesia	KJ415544	KJ415402	KJ415448
*C. cymbopogonis*	CBS 419.78	*Yucca* sp.	Netherlands	HG778985	HG779129	–
*C. dactyloctenicola*	CPC 28810 ^T^	*Dactyloctenium aegyptium*	Thailand	MF490815	MF490837	MF490858
*C. dactyloctenii*	BRIP 12846 ^T^	*Dactyloctenium radulans*	Australia	KJ415545	KJ415401	KJ415447
*C. ellisii*	CBS 193.62^T^	air	Pakistan	JN192375	JN600963	JN601007
*C. eragrostidis*	CBS 189.48	*Sorghum* sp.	Indonesia	HG778986	HG779154	–
***C. eragrosticola* sp. nov.**	BRIP 12538 ^T^	*Eragrostis pilosa*	Australia	**MH414899**	**MH433643**	**MH433661**
*C. geniculata*	CBS 187.50	*Andropogon sorghum*	Indonesia	KJ909781	KM083609	KM230410
*C. gladioli*	CBS 210.79	*Gladiolus* sp.	Romania	HG778987	HG779123	–
*C. graminicola*	BRIP 23186 ^T^	*Aristida ingrata*	Australia	JN192376	JN600964	JN601008
*C. harveyi*	BRIP 57412 ^T^	*Triticum aestivum*	Australia	KJ415546	KJ415400	KJ415446
*C. hawaiiensis*	BRIP 11987 ^T^	*Oryza sativa*	USA	KJ415547	KJ415399	KJ415445
*C. heteropogonicola*	BRIP 14579 ^T^	*Heteropogon contortus*	India	KJ415548	KJ415398	KJ415444
*C. heteropogonis*	CBS 284.91 ^T^	*Heteropogon contortus*	Australia	KJ415549	JN600969	JN601013
*C. hominis*	CBS 136985 ^T^	*Homo sapiens*	USA	HG779011	HG779106	
*C. homomorpha*	CBS 156.60 ^T^	air	USA	JN192380	JN600970	JN601014
*C. inaequalis*	CBS 102.42 ^T^	soil	France	KJ922375	KM061787	KM196574
*C. intermedia*	CBS 334.64	*Avena versicolor*	USA	HG778991	HG779155	–
*C. ischaemi*	CBS 630.82 ^T^	*Ischaemum indicum*	Solomon Islands	JX256428	JX276440	–
***C. kenpeggii* sp. nov.**	BRIP 14530 ^T^	*Triticum aestivum*	Australia	**MH414900**	**MH433644**	**MH433662**
*C. kusanoi*	CBS 137.29	*Eragrostis major*	Japan	JN192381	–	JN601016
***C. lamingtonensis* sp. nov.**	BRIP 12259 ^T^	*Microlaena stipoides*	Australia	**MH414901**	**MH433645**	**MH433663**
*C. lunata*	CBS 730.96 ^T^	*Homo sapiens*	USA	JX256429	JX276441	JX266596
*C. malina*	CBS 131274 ^T^	*Zoysia matrella*	USA	JF812154	KP153179	KR493095
***C. mebaldsii* sp. nov.**	BRIP 12900 ^T^	*Cynodon transvaalensis*	Australia	**MH414902**	**MH433647**	**MH433664**
	BRIP 13983	*Cynodondactylon x transvaalensis*	Australia	**MH414903**	**MH433646**	**MH433665**
*C. miyakei*	CBS 197.29 ^T^	*Eragrostis pilosa*	Japan	KJ909770	KM083611	KM196568
*C. muehlenbeckiae*	CBS 144.63 ^T^	*Sorghum* sp.	USA	KP400647	KP419996	KM196578
*C. neergaardii*	BRIP 12919 ^T^	*Oryza sativa*	Ghana	KJ415550	KJ415397	KJ415443
*C. neoindica*	IMI 129790 ^T^	*Brassica nigra*	India	**MH414910**	**MH433649**	**MH433667**
*C. nicotiae*	BRIP 11983 ^T^	soil	Algeria	KJ415551	KJ415396	KJ415442
*C. nodosa*	CPC 28800 ^T^	*Digitaria ciliaris*	Thailand	MF490816	MF490838	MF490859
*C. nodulosa*	CBS 160.58	*Eleusine indica*	USA	JN601033	JN600975	JN601019
*C. oryzae*	CBS 169.53 ^T^	*Oryza sativa*	Vietnam	KP400650	KP645344	KM196590
*C. ovariicola*	CBS 470.90 ^T^	*Eragrostis interrupta*	Australia	JN192384	JN600976	JN601020
*C. pallescens*	CBS 156.35 ^T^	air	Indonesia	KJ922380	KM083606	KM196570
*C. papendorfii*	CBS 308.67 ^T^	*Acacia karroo*	South Africa	KJ415552	KJ415395	KJ415441
***C. petersonii* sp. nov.**	BRIP 14642 ^T^	*Dactyloctenium aegyptium*	Australia	**MH414905**	**MH433667**	**MH433668**
*C. perotidis*	CBS 350.90 ^T^	*Perotis rara*	Australia	JN192385	KJ415394	JN601021
*C. pisi*	CBS 190.48 ^T^	*Pisum sativum*	Canada	KY905678	KY905690	KY905697
***C. platzii* sp. nov.**	BRIP 27703b ^T^	*Cenchrus clandestinus*	Australia	**MH414906**	**MH433651**	**MH433669**
*C. portulacae*	BRIP 14541 ^T^	*Portulaca oleracea*	USA	KJ415553	KJ415393	KJ415440
*C. prasadii*	CBS 143.64 ^T^	*Jasminum sambac*	India	KJ922373	KM061785	KM230408
*C. protuberata*	CBS 376.65 ^T^	*Deschampsia flexuosa*	UK	KJ922376	KM083605	KM196576
*C. pseudobrachyspora*	CPC 28808 ^T^	*Eleusine indica*	Thailand	MF490819	MF490841	MF490862
*C. pseudolunata*	UTHSC 09-2092 ^T^	*Homo sapiens*	USA	HE861842	HE861842	–
*C. pseudorobusta*	UTHSC 08-3458	*Homo sapiens*	USA	HE861838	HF565476	–
*C. ravenelii*	BRIP 13165 ^T^	*Sporobolus fertilis*	Australia	JN192386	JN600978	JN601024
***C. reesii* sp. nov.**	BRIP 4358 ^T^	air	Australia	**MH414907**	**MH433637**	**MH433670**
*C. richardiae*	BRIP 4371 ^T^	*Richardia brasiliensis*	Australia	KJ415555	KJ415391	KJ415438
*C. robusta*	CBS 624.68 ^T^	*Dichanthium annulatum*	USA	KJ909783	KM083613	KM196577
*C. ryleyi*	BRIP 12554 ^T^	*Sporobolus creber*	Australia	KJ415556	KJ415390	KJ415437
*C. senegalensis*	CBS 149.71	unknown	Nigeria	HG779001	HG779128	–
*C. soli*	CBS 222.96^T^	soil	Papua New Guinea	KY905679	KY905691	KY905698
*C. sorghina*	BRIP 15900 ^T^	*Sorghum bicolor*	Australia	KJ415558	KJ415388	KJ415435
*C. spicifera*	CBS 274.52	soil	Spain	JN192387	JN600979	JN601023
***C. sporobolicola* sp. nov.**	BRIP 23040b ^T^	*Sporobolus australasicus*	Australia	**MH414908**	**MH433652**	**MH433671**
*C. subpapendorfii*	CBS 656.74 ^T^	soil	Egypt	KJ909777	KM061791	KM196585
*C. trifolii*	CBS 173.55	*Trifolium repens*	USA	HG779023	HG779124	–
*C. tripogonis*	BRIP 12375 ^T^	*Tripogon loliiformis*	Australia	JN192388	JN600980	JN601025
*C. tropicalis*	BRIP 14834 ^T^	*Coffea arabica*	India	KJ415559	KJ415387	KJ415434
*C. tsudae*	ATCC 44764 ^T^	*Chloris gayana*	Japan	KC424596	KC747745	KC503940
*C. tuberculata*	CBS 146.63 ^T^	*Zea mays*	India	JX256433	JX276445	JX266599
*C. uncinata*	CBS 221.52 ^T^	*Oryza sativa*	Vietnam	HG779024	HG779134	–
*C. variabilis*	CPC 28815 ^T^	*Chloris barbata*	Thailand	MF490822	MF490844	MF490865
*C. verruciformis*	CBS 537.75	*Vanellus miles*	New Zealand	HG779026	HG779133	–
*C. verruculosa*	CBS 150.63	*Punica granatum*	India	KP400652	KP645346	KP735695
***C. warraberensis* sp. nov.**	BRIP 14817 ^T^	*Dactyloctenium aegyptium*	Australia	**MH414909**	**MH433653**	**MH433672**
*Curvularia* sp.	BRIP 17068b	*Micraira subulifolia*	Australia	**MH414904**	**MH433648**	**MH433666**
	BRIP 17439	*Trianthema portulacastrum*	Australia	AF081449	AF081406	**MH445455**

^1^ATCC: American Type Culture Collection, Manassas, Virginia, USA; BRIP: Queensland Plant Pathology Herbarium, Brisbane, Australia; CBS: Westerdijk Fungal Biodiversity Institute, Utrecht, The Netherlands; CPC: cultures of Pedro Crous, housed at Westerdijk Fungal Biodiversity Institute; ICMP: International Collection of Microorganisms for Plants, Auckland, New Zealand; IMI: International Mycological Institute, CABI-Bioscience, Egham, United Kingdom; MFLUCC: Mae Fah Luang University Culture Collection, Chiang Rai, Thailand; UTHSC: Fungus Testing Laboratory, University of Texas Health Science Center, San Antonio, Texas, USA.

^T^Ex-type isolates.

GenBank accessions derived from this study are shown in **bold**.

Colonies were described from 7-d-old cultures grown on potato dextrose agar (PDA) (Becton Dickinson), incubated at room temperature (approx. 25 °C) and exposed to near-ultraviolet light on a diurnal cycle. Images of the colonies and herbarium specimens were captured by an Epson Perfection V700 scanner at a 300 dpi resolution. Colour of the colonies was rated according to Rayner (1970). Taxonomic novelties were deposited in MycoBank (http://www.MycoBank.org; Crous et al. 2004).

### DNA isolation, amplification, and phylogenetic analyses

Isolates were grown on PDA for 7 d at room temperature (approx. 25 °C). Mycelium was scraped off the PDA cultures and macerated with 0.5 mm glass beads (Daintree Scientific) in a Tissue Lyser (Qiagen). Genomic DNA was extracted with the Gentra Puregene DNA Extraction Kit (Qiagen) according to the manufacturer’s instructions. Amplification and sequencing of the internal transcribed spacer (ITS) region, glyceraldehyde-3-phosphate dehydrogenase (*gapdh*) and the translation elongation factor 1-alpha (*tef1α*) loci followed the methods by Tan et al. (2014). All sequences generated were assembled using Geneious v. 9.1.8 (Biomatters Ltd) and deposited in GenBank (Table 1, in bold). Sequences were aligned with selected sequences of *Curvularia* species obtained from GenBank (Table 1) using the MAFFT alignment algorithm (Katoh et al. 2009) in Geneious. *Bipolaris maydis* (CBS 136.29) was included as the outgroup. The sequences of each locus were aligned separately and manually adjusted where necessary. The alignment included sequences from ex-type cultures of 63 species of *Curvularia* and from the reference cultures of 16 species. The Maximum-Likelihood (ML) and Bayesian Inference (BI) methods were used in phylogenetic analyses as described by Tan et al. (2016). Briefly, the ML analysis was run using RAxML v.7.2.8 (Stamatakis and Alachiotis 2010) in Geneious and started from a random tree topology. The nucleotide substitution model used was GTR with a gamma-distributed rate variation. The Markov chain Monte Carlo (MCMC) algorithm was used to create a phylogenetic tree based on Bayesian probabilities using MrBayes v.3.2.1 (Huelsenbeck and Ronquist 2001, Ronquist and Huelsenbeck 2003) in Geneious. To remove the need for *a priori* model testing, the MCMC analysis was set to sample across the entire general time-reversible (GTR) model space with a gamma-distributed rate variation across the sites. Ten million random trees were generated using the MCMC procedure with four chains. The sample frequency was set at 100 and the temperature of the heated chain was 0.1. Burn-in was set at 25%, after which the likelihood values were stationary. The concatenated alignment was deposited in TreeBASE (S22563).

**Figure 1.**
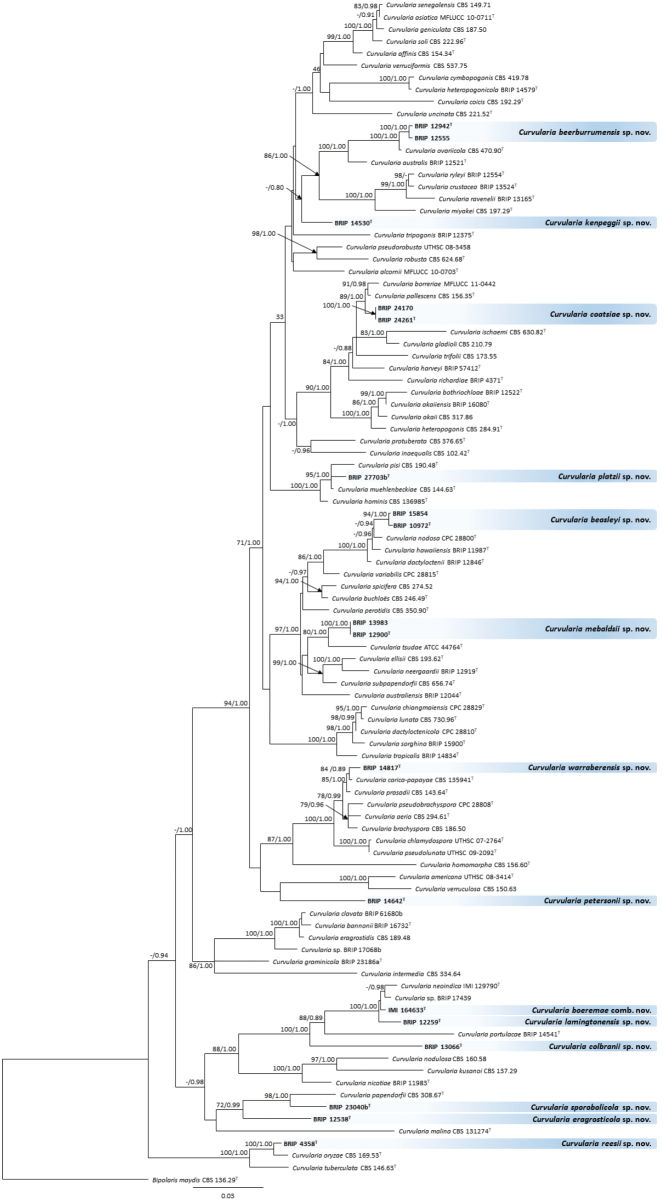
Phylogenetic tree based on maximum likelihood analysis of the combined multilocus alignment. RAxML bootstrap values (bs) greater than 70% and Bayesian posterior probabilities (pp) greater than 0.7 are given at the nodes (bs/pp). Novel species names are highlighted in blue. Ex-type isolates are marked with a T. The outgroup is *Bipolaris maydis* ex-type strain CBS 136.29.

Unique fixed nucleotide positions were used to characterise and describe two cryptic species (see applicable species notes). For each of the cryptic species that was described, the closest phylogenetic neighbour was selected (Fig. 1) and this focused dataset was subjected to single nucleotide polymorphism (SNP) analysis. These SNPs were determined for each aligned locus using the Find Variation/SNPs feature in Geneious. The SNPs were determined based on a minimum variant frequency of 0.2.

## Results

### Molecular phylogeny

Approximately 800 bp of the ITS region, 598 bp of the partial region of the *gapdh* gene and 969 bp of the partial region of the *tef1α* gene were sequenced from the BRIP isolates. After removing ambiguously aligned regions, the ITS, *gapdh* and *tef1α* alignments were trimmed to 474 bp, 544 bp and 867 bp, respectively. The ITS phylogeny was able to resolve 53 of 79 *Curvularia* species, including 10 of the new species (data not shown). The *gapdh* phylogeny inferred 12 new species and the *tef1α* phylogeny resolved all 13 of the new species (data not shown). As the topologies of the single locus phylogenies for the tree datasets did not show any conflicts, they were analysed in a concatenated alignment. The phylogenetic tree based on the concatenated alignment resolved the 17 BRIP isolates into 13 well-supported and unique clades (Fig. 1), which are described in this study as novel species.

## Taxonomy

### Curvularia beasleyi

Taxon classificationFungiPleosporalesPleosporaceae

Y.P. Tan & R.G. Shivas sp. nov.

825449

Fig. 2A–D

#### Type.

Australia, Queensland, Beaudesert, from leaf spot on *Chloris gayana*, 9 Jan. 1974, *J.L. Alcorn* (holotype BRIP 10972, includes ex-type culture).

#### Description.

*Colonies* on PDA approx. 4 cm diam. after 7 d at 25 °C, surface funiculose, margin fimbriate, olivaceous black. *Hyphae* subhyaline, smooth to branched, septate, up to 3 µm in width. *Conidiophores* branched, erect, straight to flexuous, geniculate towards apex, brown, paler towards apex, smooth, septate, up to 110 µm long, 4 µm wide; basal cell swollen and darker than the other cells, up to 6 µm diam. *Conidiogenous cells* integrated, terminal or intercalary, sympodial, pale brown, smooth, with darkened scars. *Conidia* fusiform, straight to slightly curved, rounded at the apex, (14–) 26–29 (–34) × (5–) 6.5–7.5 (–9) µm, brown to dark brown, 3–7 (mostly 5)-distoseptate; hila conspicuous, slightly protuberant, thickened and darkened, 1−1.5 µm wide.

**Figure 2.**
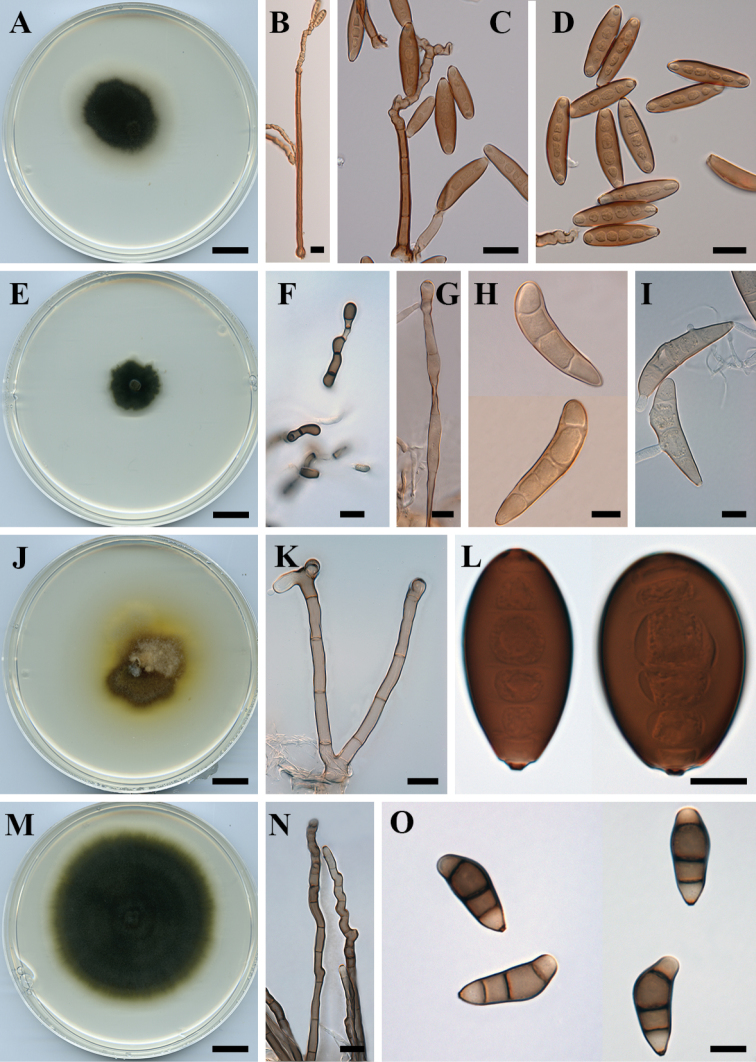
*Curvularia beasleyi* (BRIP 10972): **A** colony on PDA **B–C** conidiophores and conidia **D** conidia. *Curvularia beerburrumensis* (BRIP 12942) **E** colony on PDA **F** chlamydospores **G** conidiophore **H–I** conidia. *Curvularia boeremae* (IMI 164633) **J** colony on PDA **K c**onidiophores **L** conidia. *Curvularia coatesiae* (BRIP 24261) **M** colony on PDA **N** conidiophores **O** conidia. Scale bars: 1 cm (**A, E, J, M**); all others – 10 µm.

#### Etymology.

In recognition of Dr Dean R. Beasley, an Australian plant pathologist, for his dedication and numerous innovative contributions to the curation and promotion of the Queensland Plant Pathology Herbarium (BRIP).

#### Additional material examined.

Australia, Queensland, Atherton, from leaf spot on *Leersia hexandra*, 1 May 1987, *J.L. Alcorn*, BRIP 15854 (includes culture).

#### Notes.

*Curvularia beasleyi* is placed in the same clade as *C. dactyloctenii*, *C. hawaiiensis* and *C. nodosa* (Fig. 1). *Curvularia dactyloctenii* and *C. hawaiiensis* have been recorded in Australia (Sivanesan 1987, Tan et al. 2014), but the recently described *C. nodosa* has only been reported from Thailand (Marin-Felix et al. 2017b). *Curvularia beasleyi* is distinguished in two loci from the ex-type cultures of *C. dactyloctenii* (99% in *gapdh* and 99% in *tef1α*), *C. hawaiiensis* (98% in *gapdh* and 99% in *tef1α*) and *C. nodosa* (99% in *gapdh* and 99% in *tef1α*). The conidia of *C. beasleyi* are longer than those of *C. nodosa* (12–25 µm, Marin-Felix et al. 2017b) and shorter than those of *C. dactyloctenii* (32–55 µm, Sivanesan 1987). *Curvularia beasleyi* is morphologically similar to *C. hawaiiensis*, however the later species has never been recorded on *Leersia* (Farr and Rossman 2018).

*Curvularia beasleyi* is only known from Queensland on two unrelated grasses, the introduced host *Chloris gayana* and the native *Leersia hexandra*. There are many *Curvularia* species reported as associated with *Chloris* spp. (*C. australiensis*, *C. australis, C. hawaiiensis*, *C. lunata*, *C. nodosa*, *C. pallescens*, *C. tsudae*, *C. variabilis*, *C. verruculosa*) (Sivanesan 1987, Deng et al. 2014, Manamgoda et al. 2014, Marin-Felix et al. 2017b) and *Leersia* spp. (*C. australiensis*, *C. geniculata*, and *C. heteropogonicola*) (DAF Biological Collections 2018, Farr and Rossman 2018, Herbarium Catalogue 2018), although not all of the reports have been verified by molecular phylogenetic analyses.

### Curvularia beerburrumensis

Taxon classificationFungiPleosporalesPleosporaceae

Y.P. Tan & R.G. Shivas sp. nov.

825450

Fig. 2E–I

#### Type.

Australia, Queensland, Beerburrum, from blackened inflorescence of *Eragrostis bahiensis*, 24 May 1979, *J.L. Alcorn* (holotype BRIP 12942, includes ex-type culture).

#### Description.

*Colonies* on PDA approx. 2 cm diam. after 7 d at 25 °C, surface funiculose, margin fimbrillate, olivaceous black. *Hyphae* subhyaline, smooth to asperulate, branched, septate, 3−4 µm in width; chlamydospores intercalary in chains, 4–9 µm, smooth, thick-walled. *Conidiophores* erect, straight to flexuous, geniculate towards apex, subhyaline to pale brown, smooth, septate, up to 500 µm long, 5−6 µm wide. *Conidiogenous cells* integrated, terminal or intercalary, with sympodial proliferation, pale brown to brown, smooth, mono- or polytretic, with darkened scars. *Conidia* fusiform to subcylindrical or clavate, straight to slightly curved, rounded at the apex, (40–) 51–56 (–71) × (10–) 12–13 (–14) µm, subhyaline to pale yellowish-brown, 2–4 (mostly 3)-distoseptate; hila mostly inconspicuous or minutely thickened and darkened.

#### Etymology.

Named after the town Beerburrum, where the holotype was collected.

#### Additional material examined.

Australia, Queensland, Beerburrum, New South Wales, Yetman, blackened inflorescence of *Eragrostis sororia*, 12 May 1977, *J.L. Alcorn*, BRIP 12555 (includes culture).

#### Notes.

*Curvularia beerburrumensis* is phylogenetically sister to *C. australis* and *C. ovariicola* (Fig. 1), which have both been recorded in Australia on *Eragrostis* (Sivanesan 1987, Tan et al. 2014). *Curvularia beerburrumensis* is distinguished from the ex-type culture of *C. australis* in three loci (98% in ITS, 96% in *gapdh* and 98% in *tef1α*). Furthermore, *C. beerburrumensis* has larger conidia than *C. australis* (25−48 × 9.0−12.5 µm, Sivanesan 1987). *Curvularia beerburrumensis* differs from the ex-type culture of *C. ovariicola* in three loci (99% in ITS, 99% in *gapdh* and 99% in *tef1α*). *Curvularia beerburrumensis* has longer conidiophores than *C. ovariicola* (up to 325 µm, Sivanesan 1987). *Curvularia beerburrumensis* also produced chlamydospores in culture, which are not known for *C. australis* and *C. ovariicola*.

*Curvularia beerburrumensis* is only known from inflorescences of the invasive South American grass *Eragrostis bahiensis*, as well as the Australian native *E. sororia* (Simon and Alfonso 2011). Other *Curvularia* associated with *Eragrostis* include *C. australis*, *C. clavata*, *C. crustacea*, *C. ellisii*, *C. eragrostidis*, *C. geniculata*, *C. kusanoi*, *C. lunata*, *C. miyakei*, *C. nodulosa*, *C. ovariicola*, *C. perotidis*, *C. protuberata*, *C. ravenelii* and *C. verrucosa*, (Sivanesan 1987, Farr and Rossman 2018, Herbarium Catalogue 2018), although many of these reports are yet to be verified by molecular phylogenetic analyses.

### Curvularia boeremae

Taxon classificationFungiPleosporalesPleosporaceae

(A.S. Patil & V.G. Rao) Y.P. Tan & R.G. Shivas comb. nov.

825451

Fig. 2J–L

#### Basionym.

*Drechslera boeremae* A.S. Patil & V.G. Rao, *Antonie van Leeuwenhoek* 42: 129 (1976).

#### Description.

*Colonies* on PDA approx. 3 cm diam. after 7 d at 25 °C, surface funiculose, margin fimbriate, olivaceous green to citrine, velutinous with aerial mycelium. *Hyphae* subhyaline, smooth to asperulate, branched, septate, 2–3 µm in width. *Conidiophores* straight to flexuous, slightly geniculate towards apex, uniformly subhyaline to pale brown, smooth, septate, up to 110 µm long, 4 µm wide. *Conidiogenous cells* integrated, terminal or intercalary, with sympodial proliferation, pale brown to brown, smooth, mono- or polytretic, with darkened scars. *Conidia* broadly ellipsoidal to oval, brown to dark brown, smooth, (42–) 46–52 (–55) × (17–) 20–23 (–25) µm, brown to dark brown, 4–6-distoseptate, hila protuberant, thickened and darkened, 2–3 µm wide.

#### Type.

India, Poona, from leaves of *Portulaca oleracea*, 28 Apr. 1970, *A.S. Patil* (holotype IMI 164633, includes ex-type culture), (isotype BRIP 13934, includes ex-type culture).

#### Notes.

Multilocus phylogenetic analyses placed the ex-type culture of *D. boeremae* within the clade that includes *C. lunata*, the type species of the genus (Fig. 1). *Curvularia boeremae* differs from *C. neoindica* in one locus (98% identities in *gapdh*). Furthermore, *C. boeremae* has shorter conidia than *C. neoindica* (27–65 µm, Manamgoda et al. 2014). Sivanesan’s (1987) synonymy of *Drechslera boeremae* with *Bipolaris indica* was based on similar conidial morphology and is not supported by the phylogenetic analyses in this study.

*Curvularia boeremae* is only known from the type specimen on *P. oleraceae* and has not been recorded in Australia. *Curvularia portulacae* is the only other species recorded on *P. oleraceae* (Farr and Rossman 2018). *Curvularia boeremae* is morphologically distinct from *C. portulacae*, which has comparatively long, cylindrical conidia (average 110 × 13 µm, Rader 1948).

### Curvularia coatesiae

Taxon classificationFungiPleosporalesPleosporaceae

Y.P. Tan & R.G. Shivas sp. nov.

825452

Fig. 2M–O

#### Type.

Australia, Queensland, Eudlo, from rotted fruit of *Litchi chinensis*, 28 Jan. 1992, *L.M. Coates* (holotype BRIP 24261, includes ex-type culture).

#### Description.

*Colonies* on PDA 6–7 cm diam. after 7 d at 25 °C, surface funiculose, floccose, olivaceous black at the centre, olivaceous to grey olivaceous towards the edge, margin fimbriate. *Hyphae* subhyaline, smooth to asperulate, septate, up to 3 µm in width. *Conidiophores* erect, flexuous, geniculate in the top half, uniformly brown, sometimes pale towards apex, septate, up to 190 µm long, 4 µm wide; basal cell sometimes swollen, up to 8 µm diam. *Conidiogenous cells* integrated, terminal or intercalary, with sympodial proliferation, pale brown, mono- or polytretic, with darkened nodes. *Conidia* ellipsoidal to obovoid, asymmetrical, sometimes the third cell from base is unequally enlarged, intermediate cells dark brown and usually verruculose, end cells paler and less ornamented than central cells, (20–) 23–26 (–30) × (7–) 8–9 (–10) µm, 3-distoseptate; hila protuberant, thickened and darkened, 1–2 µm wide.

#### Etymology.

Named after Dr Lindel (Lindy) M. Coates, an Australian plant pathologist in recognition of her contributions to the study of post-harvest fruit pathology.

#### Additional material examined.

Australia, New South Wales, Alstonville, isolated from the air in a mango orchard, 11 Mar. 1991, *G.I. Johnson*, BRIP 24170 (includes culture).

#### Notes.

*Curvularia coatesiae* is morphologically similar and phylogenetically related to a reference culture of *C. borreriae* and the ex-type culture of *C. pallescens* (Fig. 1). *Curvularia coatesiae* differs from the ex-type culture of *C. pallescens* in three loci: ITS position 439 (T); *gapdh* positions 219 (C), 287 (C); *tef1α* positions 43 (C), 257 (C), 259 (C). Although *C. borreriae* and *C. pallescens* have been recorded in Australia, these have not been verified by molecular phylogenetic analyses and there have been no additional records beyond the 1980s (Sivanesan 1987, Shivas 1989). Other species recorded from *L. chinensis* are *C. geniculata*, *C. hawaiiensis*, *C. lunata* and *C. pallescens* (DAF Biological Collections 2018, Herbarium Catalogue 2018), although not all the reports have been verified by molecular phylogenetic analyses.

### Curvularia colbranii

Taxon classificationFungiPleosporalesPleosporaceae

Y.P. Tan & R.G. Shivas sp. nov.

825453

Fig. 3A–D

#### Type.

Australia, Queensland, Brisbane, from leaf spot on *Crinum zeylanicum*, 11 Oct. 1976, *R.C. Colbran* (holotype BRIP 13066, includes ex-type culture).

#### Description.

*Colonies* on PDA approx. 5 cm diam. after 7 d at 25 °C, surface funiculose, margin fimbriate, olivaceous black, aerial mycelium white. *Hyphae* subhyaline, smooth, septate, up to 3 µm in width. *Conidiophores* erect, flexuous, geniculate, uniformly pale brown to brown, smooth, septate, up to 145 µm long, 4–6 µm wide, basal cell sometimes swollen, up to 8 µm diam. *Conidiogenous cells* integrated, terminal or intercalary, with sympodial proliferation, pale brown to brown, smooth, mono- or polytretic, with darkened scars. *Conidia* fusiform to subcylindrical with rounded apex and obconical at the base, brown, end cells pale, (54–) 83–92 (–110) × (13–) 14–16 (–17) µm, brown to dark brown, 6–9-distoseptate; hila slightly protuberant, thickened and darkened, 1–2 µm wide.

**Figure 3.**
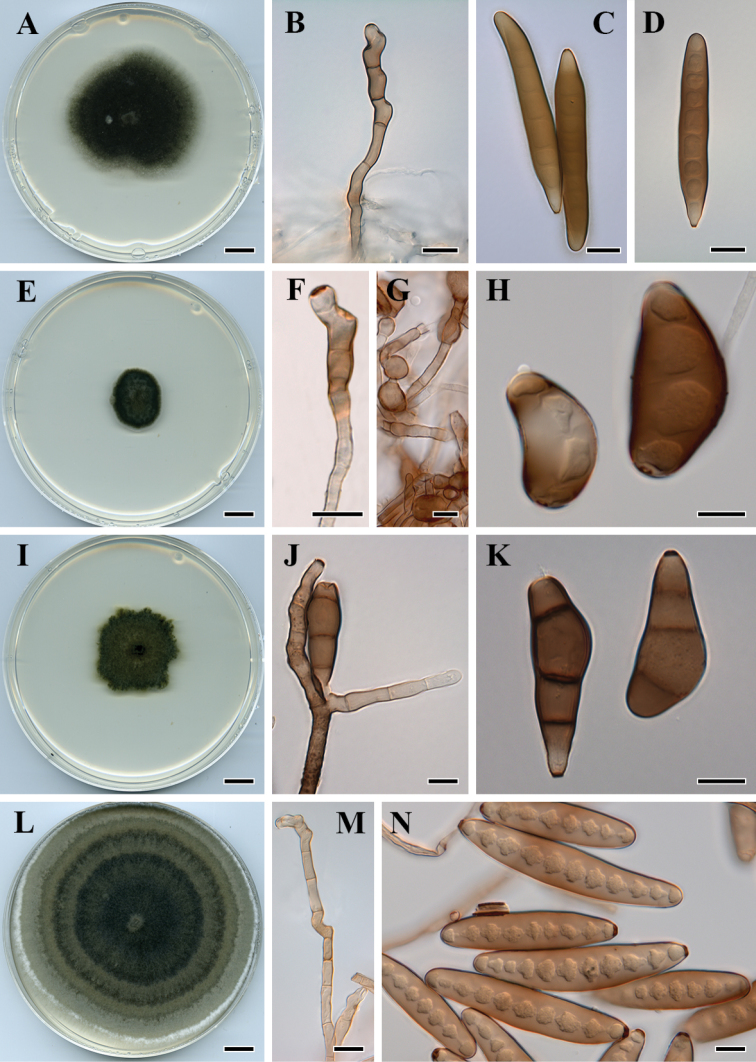
*Curvularia colbranii* (BRIP 13066): **A** colony on PDA **B** conidiophore **C–D** conidia. *Curvularia eragrosticola* (BRIP 12538) **E** colony on PDA **F** conidiophore **G** chlamydosphores **H** conidia. *Curvularia kenpeggii* (BRIP 14530) **I** colony on PDA **J** conidiophores and conidium **K** conidia. *Curvularia lamingtonensis* (BRIP 12259) **L** colony on PDA **M** conidiophore **O** conidia. Scale bars: 1 cm (**A, E, I, L**); all others – 10 µm.

#### Etymology.

Named after Dr Robert (Bob) Chester Colbran (1926–2010), an Australian nematologist and Director of the Plant Pathology Branch, Queensland Department of Primary Industries, in recognition of his significant contributions to Australian plant pathology.

#### Notes.

*Curvularia colbranii* is sister to *C. boeremae*, *C. lamingtonensis* (see this paper), *C. neoindica* and *C. portulacae*, although separated by a considerable genetic distance (Fig. 1). *Curvularia colbranii* has fusiform to subcylindrical conidia that are distinct from the ellipsoidal to oval conidia of *C. boeremae* (42–55 × 17–25 µm, this study) and *C. neoindica* (27–65 × 17–27 µm, Manamgoda et al. 2014) and longer than those of *C. lamingtonensis* (45–76 × 11–14 µm, this study). *Curvularia colbranii* has conidia that are 6–9-distoseptate, while *C. portulacae* has conidia reported as 3–15 celled (Rader 1948).

Only one other species, *C. trifolii*, has been reported on *Crinum* sp. (Shaw 1984), but this record has not been verified by phylogenetic analyses. *Curvularia colbranii* is morphologically distinct from *C. trifolii*, which has curved conidia.

### Curvularia eragrosticola

Taxon classificationFungiPleosporalesPleosporaceae

Y.P. Tan & R.G. Shivas sp. nov.

825454

Fig. 3E–H

#### Type.

Australia, New South Wales, Yetman, from inflorescence on *Eragrostis pilosa*, 12 May 1977, *J.L. Alcorn* (holotype BRIP 12538, includes ex-type culture).

#### Description.

*Colonies* on PDA approx. 2 cm diam. after 7 d at 25 °C, surface funiculose, margin fimbriate, dark olive with white patches, velutinous with some aerial mycelium. *Hyphae* subhyaline, smooth, branched, septate, 4−5 µm wide; chlamydospores abundant, subglobose to ellipsoidal or irregular, terminal and intercalary, 5−20 µm diam. *Conidiophores* erect, straight to flexuous, slightly geniculate, pale brown to brown, paler towards apex smooth, septate, up to 145 µm long, 4−5 µm wide. *Conidiogenous cells* integrated, terminal or intercalary, sympodial, pale brown to brown, smooth, with darkened scars. *Conidia* hemi-ellipsoidal, curved, asymmetrical, brown to dark brown, end cells slightly paler, (25–) 26–30 (–34) × (9–) 13–15 (–19) µm, 3-distoseptate with a faint narrow median septum; hila non-protuberant, minutely thickened and darkened.

#### Etymology.

Named after *Eragrostis*, the grass genus from which this fungus was isolated.

#### Notes.

*Curvularia eragrosticola* is phylogenetically close to *C. papendorfii* and *C. sporobolicola* (see this paper) (Fig. 1). *Curvularia eragrosticola* is distinguished in three loci from the ex-type culture of *C. papendorfii* (97% in ITS, 92% in *gapdh* and 98% in *tef1α*) and *C. sporobolicola* (98% in ITS, 92% in *gapdh* and 98% in *tef1α*). *Curvularia eragrosticola* has conidia that are smaller than *C. papendorfii* (30–50 × 17–30 µm, Sivanesan 1987) and *C. sporobolicola* (34–45 × 14–23 µm, this study).

*Curvularia eragrosticola* is only known from the type specimen on *Eragrostis pilosa*, which is native to Eurasia and Africa and is considered a troublesome weed in Australia (Simon and Alfonso 2011). Neither *C. papendorfii* nor *C. sporobolicola* have been reported on *Eragrostis*. Other *Curvularia* spp. associated with *Eragrostis* are listed in the notes for *C. beerburrumensis*.

### Curvularia kenpeggii

Taxon classificationFungiPleosporalesPleosporaceae

Y.P. Tan & R.G. Shivas sp. nov.

825455

Fig. 3H–J

#### Type.

Australia, Queensland, from mouldy grain of *Triticum aestivum*, 26 Oct. 1984, *J.L. Alcorn* (holotype BRIP 14530, includes ex-type culture), (isotype IMI 290719).

#### Description.

*Colonies* on PDA 3–4 cm diam. after 7 d at 25 °C, surface funiculose, margin fimbriate, floccose and olivaceous black at the centre with white patches, velutinous with some aerial mycelium. *Hyphae* hyaline, asperulate, branched, septate, 4−5 µm in width. *Conidiophores* erect, straight to flexuous, slightly geniculate in the upper part, pale brown to brown, sometimes paler towards the apex, verrucose, septate, up to 360 µm long, 4−5 µm wide, basal cell sometimes swollen, up to 8 µm. *Conidiogenous cells* integrated, terminal or intercalary, with sympodial proliferation, pale brown to brown, smooth, mono- or polytretic, with darkened scars. *Conidia* ellipsoidal to clavate to obovoid, asymmetrical, third cell from the base is unequally enlarged, brown, end cells paler, verruculose, (31–) 35–39 (–42) × (10–) 13–14 (–15) µm, 3-distoseptate, hila protuberant, thickened and darkened, 1–2 µm wide.

#### Etymology.

Named after Dr Kenneth G. Pegg AM (member of the Order of Australia), in celebration of his 60 years of dedication to plant pathology in Australia and to thank him for his generous mentorship.

#### Notes.

*Curvularia kenpeggii* is only known from the holotype specimen and is genetically distinct from all other *Curvularia* species (Fig. 1). *Curvularia kenpeggii* is basal to a clade comprised of *C. australis*, *C. beerburrumensis*, *C. crustacea*, *C. miyakei*, *C. ovariicola*, *C. ravenelii* and *C. ryleyi*. These species are mostly reported as pathogens of *Eragrostis* and *Sporobolus* spp. and not known to be associated with wheat (*Triticum aestivum*). *Curvularia* species associated with *T. aestivum* in Australia are *C. brachyspora*, *C. harveyi*, *C. hawaiiensis*, *C. lunata*, *C. perotidis*, *C. ramosa* and *C. spicifera*, (Shivas 1989, Farr and Rossman 2018), although not all the reports have been verified by molecular phylogenetic analyses.

### Curvularia lamingtonensis

Taxon classificationFungiPleosporalesPleosporaceae

Y.P. Tan & R.G. Shivas sp. nov.

825456

Fig. 3L–N

#### Type.

Australia, Queensland, Lamington National Park, from *Microlaena stipoides*, 09 May 1977, *J.L. Alcorn* (holotype BRIP 12259, includes ex-type culture).

#### Description.

*Colonies* on PDA cover the whole plate after 7 d at 25 °C, surface funiculose, margin fimbriate, olivaceous green, velutinous with some aerial mycelium. *Hyphae* hyaline, branched, septate, 4 µm in width. *Conidiophores* erect, straight to flexuous, geniculate towards apex, pale brown to dark brown on wheat straw agar, septate, up to 160 µm long, 3−4 µm wide. *Conidiogenous cells* integrated, terminal or intercalary, sympodial, pale brown to brown, smooth, with darkened scars. *Conidia* ellipsoidal to fusiform, straight, pale brown, (45–) 59–66 (–76) × (11–) 11.5–13 (–14) µm, 4–11-distoseptate with inconspicuous transverse septa, hila protuberant, thickened and darkened, 1–2 µm wide.

#### Etymology.

Named after the locality, Lamington National Park, where the holotype was collected.

#### Notes.

*Curvularia lamingtonensis* is phylogenetically closely related to *C. boeremae* and *C. neoindica*. *Curvularia lamingtonensis* is distinguished from the ex-type culture of *C. boeremae* in two loci (96% in ITS and 98% in *gapdh*) and from the ex-type culture of *C. neoindica* in three loci (95% in ITS, 98% in *gapdh* and 99% in *tef1α*). *Curvularia lamingtonensis* has longer and straighter conidia than *C. boeremae* and *C. neoindica*, both of which have broad, ellipsoidal conidia (42–55 × 20–23 µm, and 27–65 × 17–27 µm, respectively). *Curvularia lamingtonensis* is only known from the type specimen on *Microlaena stipoides*. This is the first record of a *Curvularia* species associated with *Microlaena*.

### Curvularia mebaldsii

Taxon classificationFungiPleosporalesPleosporaceae

Y.P. Tan & R.G. Shivas sp. nov.

825457

Fig. 4A–C

#### Type.

Australia, Victoria, Hopetoun, from *Cynodon transvaalensis*, Apr. 1979, *M. Mebalds* (holotype BRIP 12900, includes ex-type culture).

#### Description.

*Colonies* on PDA approx. 5 cm diam. after 7 d at 25 °C, surface funiculose, margin fimbriate, olivaceous black with white patches, velutinous with some aerial mycelium. *Hyphae* hyaline to subhyaline, smooth to asperulate, septate, 3–4 µm wide. *Conidiophores* erect, straight to flexuous, sometimes slightly geniculate towards apex, branched, uniformly brown, paler at apex, smooth to asperulate, septate, up to 180 µm long, 4–5 µm wide. *Conidiogenous cells* integrated, terminal or intercalary, with sympodial proliferation, subhyaline to pale brown, smooth, mono- or polytretic, with darkened scars. *Conidia* ellipsoidal to obovoid, sometimes straight to slightly curved, rounded at the apex, (22–) 25–28 (–30) × (7–) 8–9 (–10) µm, pale brown to brown, 3-distoseptate, hila protuberant, thickened and darkened, 1–2 µm wide.

**Figure 4.**
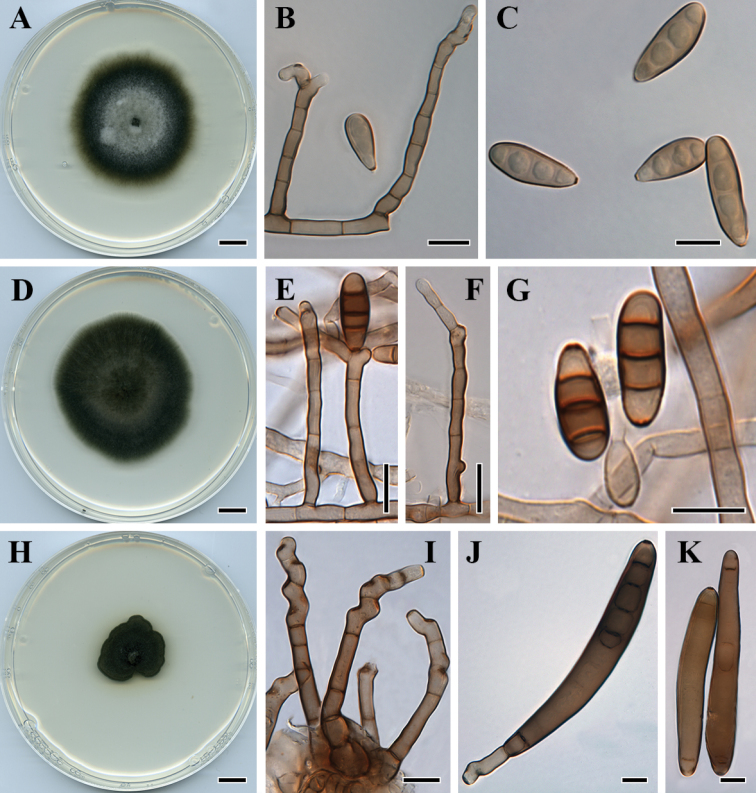
*Curvularia mebaldsii* (BRIP 12900): **A** colony on PDA **B** conidiophores and conidium **C** conidia. *Curvularia petersonii* (BRIP 14642) **D** colony on PDA **E–F** conidiophores and conidium **G** conidia. *Curvularia platzii* (BRIP 27703b) **H** colony on PDA **I** conidiophores **J–K** conidia. Scale bars: 1 cm (**A, D, H**); all others – 10 µm.

#### Etymology.

Named after the collector, Martin Mebalds, in recognition of his contributions to Australian plant pathology and biosecurity.

#### Additional material examined.

Australia, New South Wales, Tweed Heads, from necrotic leaf on Cynodon dactylon × transvaalensis, 10 Jun. 1983, *G. Thomas*, BRIP 13983 (includes culture).

#### Notes.

The multilocus phylogenetic analyses showed that *C. mebaldsii* was sister to *C. tsudae*, although separated by a considerable genetic distance (Fig. 1). *Curvularia mebaldsii* is distinguished from the ex-type culture of *C. tsudae* in three loci (98% in ITS, 97% in *gapdh* and 99% in *tef1α*). Morphologically, *C. mebaldsii* cannot be reliably separated from *C. tsudae*.

*Curvularia mebaldsii* is known from two specimens on *Cynodon* spp. Several *Curvularia* species have been associated with *Cynodon*, including *C. aeria*, *C. australiensis*, *C. brachyspora*, *C. clavata*, *C. fallax*, *C. geniculata*, *C. hawaiiensis*, *C. inaequalis*, *C. lunata*, *C. pallescens*, *C. ramosa*, *C. senegalensis*, *C. spicata*, *C. spicifera* and *C. verruculosa* (DAF Biological Collections 2018, Farr and Rossman 2018, Herbarium Catalogue 2018), although these records have not been verified by phylogenetic analyses.

### Curvularia petersonii

Taxon classificationFungiPleosporalesPleosporaceae

Y.P. Tan & R.G. Shivas sp. nov.

825458

Fig. 4D–G

#### Type.

Australia, Northern Territory, Daly Waters, from leaf spot on *Dactyloctenium aegyptium*, 20 Mar. 1985, *R.A. Peterson* (holotype BRIP 14642, includes ex-type culture).

#### Description.

*Colonies* on PDA approx. 5 cm diam. after 7 d at 25 °C, surface funiculose, olivaceous black, velutinous with some aerial mycelium, margin fimbriate. *Hyphae* subhyaline, smooth to asperulate, septate, up to 3 µm in width. *Conidiophores* erect, straight to flexuous, rarely branched, slightly geniculate, uniformly brown, sometimes pale brown at apex, smooth, septate, up to 110 µm long, 4 µm wide. *Conidiogenous cells* integrated, terminal or intercalary, with sympodial proliferation, pale brown to brown, smooth, mono- or polytretic, with darkened scars. *Conidia* obovoid to ellipsoidal, straight to slightly curved, (15–) 17–19 (–21) × (5–) 5.5–6 (–7) µm, brown, end cells pale, 3-distoseptate, hila non-protuberant, thickened and darkened.

#### Etymology.

Named after Ron A. Peterson, an Australian plant pathologist, in recognition of his contributions to tropical plant pathology.

#### Notes.

The multilocus phylogenetic analyses placed *C. petersonii* sister to *C. americana* and *C. verruculosa*, although separated by a considerable genetic distance (Fig. 1). Both *C. americana* and *C. verruculosa* have been found in Australia (DAF Biological Collections 2018, Herbarium Catalogue 2018). *Curvularia petersonii* is distinguished from the ex-type culture of *C. americana* in two loci (94% in ITS and 92% in *gapdh*) and from a reference culture of *C. verruculosa* in three loci (92% in ITS, 92% in *gapdh* and 98% in *tef1α*). *Curvularia petersonii* has smaller conidia than *C. americana* (13–28 × 7–15 µm, Madrid et al. 2014) and *C. verruculosa* (20–40 × 12–17 µm, Sivanesan 1987).

*Curvularia petersonii* is only known from a single specimen on *Dactyloctenium aegyptium* in the Northern Territory. Many *Curvularia* species have been associated with *Dactyloctenium*, including *C. clavata*, *C. dactyloctenicola*, *C. dactyloctenii*, *C. eragrostidis*, *C. lunata*, *C. neergaardii*, *C. pallescens* and *C. verruculosa* (Sivanesan 1987, Manamgoda et al. 2014, Farr and Rossman 2018, Herbarium Catalogue 2018, Marin-Felix et al. 2017b), although these records have not been verified by phylogenetic analyses.

### Curvularia platzii

Taxon classificationFungiPleosporalesPleosporaceae

Y.P. Tan & R.G. Shivas sp. nov.

825459

Fig. 4H–I

#### Type.

Australia, Queensland, Warwick, from leaf spot on *Cenchrus clandestinus*, 24 Jan. 2001, *G.J. Platz* (holotype BRIP 27703b, includes ex-type culture).

#### Description.

*Colonies* on PDA approx. 2 cm diam. after 7 d at 25 °C, surface dark olivaceous green. *Hyphae* subhyaline, smooth, septate, up to 3 µm wide. *Conidiophores* erect, straight to flexuous, geniculate towards apex, uniformly brown, sometimes pale brown towards apex, septate, up to 75 µm long, 5–6 µm wide, swollen at base, 8–10 µm. *Conidiogenous cells* integrated, terminal or intercalary, with sympodial proliferation, pale brown to brown, smooth, mono- or polytretic, with darkened scars. *Conidia* fusiform to narrowly clavate, brown, end cells sometimes paler, (65–) 94–105 (–115) × (11–) 12.5–13.5 (–14) µm, 9–13-distoseptate; hila non-protuberant, thickened and darkened.

#### Etymology.

Named after Gregory (Greg) J. Platz, in recognition of his contributions to Australian cereal plant pathology for the past 30 years, as well as his prowess as an international and Queensland rugby league footballer.

#### Notes.

The multilocus phylogenetic analyses indicated *C. platzii* was sister to *C. hominis*, *C. meuhlenbeckiae* and *C. pisi* (Fig. 1). *Curvularia platzii* is distinguished in one locus from the ex-type culture of *C. hominis* (97% in *tef1α*) and in two loci from the reference culture of *C. meuhlenbeckiae* (99% in *gapdh* and 99% in *tef1α*) and the ex-type culture of *C. pisi* (98% in *gapdh* and 99% in *tef1α*). *Curvularia platzii* differs from *C. hominis*, *C. meuhlenbeckiae* and *C. pisi*, which have much shorter asymmetrical conidia with fewer septa (Madrid et al. 2014, Marin-Felix et al. 2017a).

*Curvularia platzii* is only known from the holotype. The host, *Cenchrus clandestinus* (syn. *Pennisetum clandestinus*), is a perennial grass with a worldwide distribution (Simon and Alfonso 2011). Other *Curvularia* species associated with *C. clandestinus* are *C. lunata*, *C. nodulosa* and *C. trifolii* (Farr and Rossman 2018, Herbarium Catalogue 2017), although these records have not been verified by phylogenetic analyses.

### Curvularia reesii

Taxon classificationFungiPleosporalesPleosporaceae

Y.P. Tan & R.G. Shivas sp. nov.

825460

Fig. 5A–C

#### Type.

Australia, Queensland, Brisbane, isolated from air, 22 Jun. 1963, *R.G. Rees* (holotype BRIP 4358, includes ex-type culture).

#### Description.

*Colonies* on PDA approx. 6−7 cm diam. after 7 d at 25 °C, surface funiculose, greenish-grey, velutinous with some aerial mycelium, margin fimbriate. *Hyphae* hyaline, branched, septate, 3−4 µm in width. *Conidiophores* erect, straight to flexuous, slightly geniculate towards apex, pale brown to brown, sometimes paler towards the apex, septate, up to 200 µm long, 4−5 µm wide. *Conidiogenous cells* integrated, terminal or intercalary, with sympodial proliferation, pale brown to brown, smooth, mono- or polytretic, with darkened scars. *Conidia* ellipsoidal to obclavate, straight, third cell from pore swollen, brown, end cells paler, smooth, (28–) 31–35 (–39) × (10–) 12–13 (–14) µm, mostly 3 septate; hila inconspicuous, sometimes darkened.

**Figure 5.**
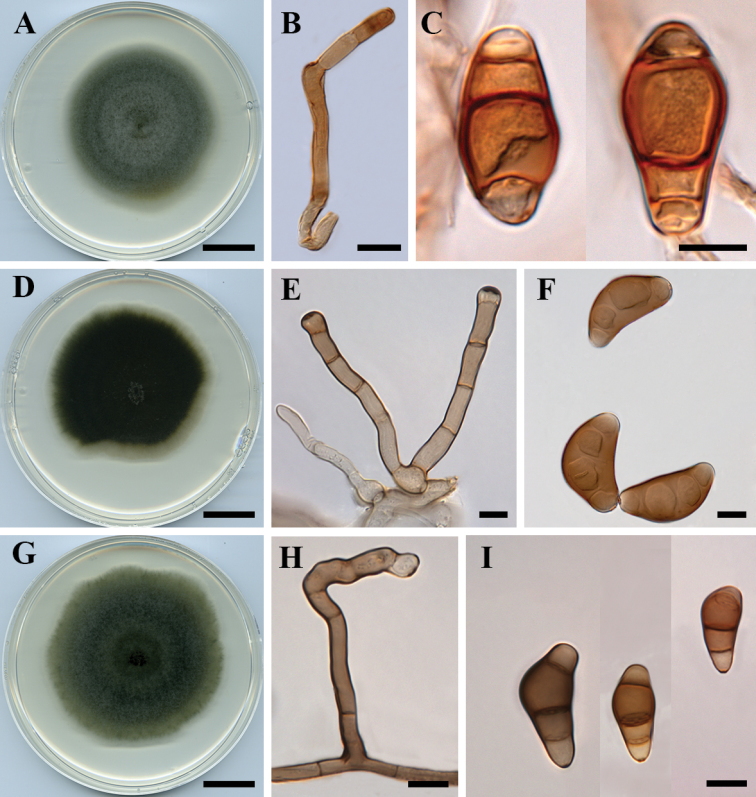
*Curvularia reesii* (BRIP 4358): **A** colony on PDA **B** conidiophore **C** conidia. *Curvularia sporobolicola* (BRIP 23040b) **D** colony on PDA **E** conidiophores **F** conidia. *Curvularia warraberensis* (BRIP 14817) **G** colony on PDA **H** conidiophore **I** conidia. Scale bars: 1 cm (**A, D, G**); all others – 10 µm.

#### Etymology.

Named after Dr Robert (Bob) G. Rees, an Australian plant pathologist, in recognition of his extensive contributions to cereal pathology.

#### Notes.

The multilocus phylogenetic analyses indicated *C. reesii* was sister to *C. oryzae* and *C. tuberculata*. *Curvularia reesii* is distinguished in two loci from the ex-type cultures of *C. oryzae* (98% in *gapdh* and 99% in *tef1α*) and *C. tuberculata* (96% in *gapdh* and 99% in *tef1α*). Morphologically, *C. reesii* has conidia similar in size to *C. oryzae* (24–40 × 12–22 µm, Sivanesan 1987) and *C. tuberculata* (23–52 × 13–20 µm, Sivanesan 1987). The isolate of *C. reesii* examined in this study had become sterile.

### Curvularia sporobolicola

Taxon classificationFungiPleosporalesPleosporaceae

Y.P. Tan & R.G. Shivas sp. nov.

825461

Fig. 5C–E

#### Type.

Australia, Queensland, Musselbrook Reserve, leaf of *Sporobolus australasicus*, 2 May 1995, *J.L. Alcorn* (holotype BRIP 23040b, includes ex-type culture).

#### Description.

*Colonies* on PDA approx. 6 cm diam. after 7 d at 25 °C, surface funiculose, margin fimbriate, olivaceous black, velutinous. *Hyphae* subhyaline, smooth, branched, septate, 3 µm wide. *Conidiophores* erect, straight to flexuous, geniculate, pale yellowish-brown, septate, up to 55 µm long, 4−5 µm wide, basal cell swollen, 6−10 µm diam. *Conidiogenous cells* cylindrical, slightly flared at the apex, integrated, sympodial, pale brown, smooth, with darkened and thickened scars. *Conidia* hemi-ellipsoidal, curved, 4-distoseptate with a faint narrow median septum, (34–) 37–41 (–45) × (14–) 17–20 (–23) µm, brown to dark brown, end cells rounded and paler, hila non-protuberant, sometimes darkened.

#### Etymology.

Named after *Sporobolus*, the grass genus from which it was isolated.

#### Notes.

Based on multilocus phylogenetic analyses, *C. sporobolicola* clustered sister to *C. papendorfii*, which are both sister to *C. eragrosticola* (Fig. 1). *Curvularia sporobolicola* is distinguished in three loci from the ex-type cultures of *C. papendorfii* (99% in ITS, 96% in *gapdh* and 98% in *tef1α*) and *C. eragrosticola* (98% in ITS, 92% in *gapdh* and 98% in *tef1α*). These three species are similar in having dark brown, hemi-ellipsoidal, curved, conidia, which makes identification by morphology difficult. The conidia of *C. sporobolicola* tend to be wider than those of C. *eragrosticola* (25–35 × 9–19 µm, this study) and *C. papendorfii* (30–50 × 9–19 µm, Sivanesan 1987).

*Curvularia sporobolicola* is only known from the type specimen on *S. australasicus*, which is a native Australian grass with a broad distribution in the tropics and subtropics (Simon and Alfonso 2011). Other *Curvularia* species associated with *Sporobolus* include *C. australis*, *C. crustacea*, *C. eragrostidis*, *C. geniculata*, *C. lunata*, *C. ovariicola*, *C. pallescens*, *C. ravenelii* and *C. ryleyi* (Sivanesan 1987, Farr and Rossman 2018), although this is the first *Curvularia* species associated with *S. australasicus*.

### Curvularia warraberensis

Taxon classificationFungiPleosporalesPleosporaceae

Y.P. Tan & R.G. Shivas sp. nov.

825462

Fig. 5F–H

#### Type.

Australia, Queensland, Torres Strait, Warraber Island, from leaf spot on *Dactyloctenium aegyptium*, 2 Jun. 1985, *R.A. Peterson* (holotype BRIP 14817, includes ex-type culture).

#### Description.

*Colonies* on PDA 6–7 mm diam. after 7 d at 25 °C, surface funiculose, margin fimbriate, olivaceous green, velutinous with some aerial mycelium. *Hyphae* subhyaline, smooth, septate, up to 3 µm wide. *Conidiophores* erect, flexuous, geniculate towards apex, uniformly pale brown to brown, septate, up to 360 µm long, 4–5 µm wide, basal cell sometimes swollen, 6–8 µm diam. *Conidiogenous cells* integrated, terminal or intercalary, with sympodial proliferation, pale brown to brown, smooth, mono- or polytretic, with darkened scars. *Conidia* ellipsoidal, curved, the third cell from base swollen, end cells paler, smooth, (20–) 23–26 (–28) × (8–) 9.5–11 µm, pale brown to brown, 3-distoseptate, hila conspicuous, sometimes slightly protuberant, thickened and darkened.

#### Etymology.

Named after the locality, Warraber Island in the Torres Straits, where the specimen was collected.

#### Notes.

Multilocus phylogenetic analyses placed *C. warraberensis* sister to *C. caricae-papayae* and *C. prasadii* (Fig. 1). *Curvularia warraberensis* differs from the ex-type culture of *C. caricae-papayae* in *gapdh* positions 40 (C), 102 (C), 230 (A), 233 (C) and 321 (A) and from the ex-type culture of *C. prasadii* in two loci, *gapdh* positions 102 (C), 131 (C), 230 (A), 233 (C), 321 (A) and *tef1α* positions 214 (C), 337 (C), 542 (A), 543 (C), 685 (C). These three species belong to the *lunata*-clade sensu Madrid et al. (2014), which also includes *C. aeria*, *C. brachyspora*, *C. chlamydospora, C. lunata* and *C. pseudolunata*. All the species in the *lunata*-clade sensu Madrid et al. (2014) have 4-celled conidia in which the third cell from the base is often swollen (unequally sided and larger) and darker than the other cells. *Curvularia warraberensis* has longer conidiophores than *C. caricae-papayae* (up to 100 µm long, Srivastava and Bilgrami 1963) and longer conidia than *C. caricae-papayae* (12.8–18.0 × 6–8 µm) and *C. prasadii* (12.8–18.0 × 6–8 µm, Mathur and Mathur 1959).

*Curvularia warraberensis* is only known from the holotype. *Curvularia* species associated with *Dactyloctenium* are listed in the notes for *C. petersonii*.

## Discussion

Although the ITS locus is the universal barcode marker for fungi (Schoch et al. 2012), secondary loci are often essential for the accurate identification of many helminthosporioid species (Manamgoda et al. 2012, 2015, Madrid et al. 2014, Tan et al. 2014, 2016, Stielow et al. 2015. Hernández-Restrepo et al. 2018). The protein-coding loci of *gapdh*, *tef1α* and RNA polymerase II second largest subunit (*rpb2*) have been reported as phylogenetically informative in the phylogenetic analyses of sequence data from species of *Curvularia* (Hernández-Restrepo et al. 2018, Manamgoda et al. 2014, Marin-Felix et al. 2017a, 2017b). In this study, sequences of three loci (ITS, *gapdh* and *tef1α*) from 17 cultures in BRIP were compared with those from ex-type cultures as well as published reference cultures for species of *Bipolaris* and *Curvularia*. The phylogenetic analyses of the concatenated three-locus dataset resolved the 17 BRIP isolates into 13 novel *Curvularia* species.

Eight *Curvularia* species are described here from grasses (Poaceae) exotic to Australia, namely, *C. beasleyi* on *Chloris gayana*, *C. beerburrumensis* on *Eragrostis bahiensis*, *C. eragrosticola* on *E. pilosa*, *C. kenpeggii* on *Triticum aestivum*, *C. mebaldsii* on *Cynodondactylon × transvaalensis*, *C. petersonii* and *C. warraberensis* on *Dactyloctenium aegyptium* and *C. platzii* on *Cenchrus clandestinus*. Only two species were described from native Australian grasses, *C. lamingtonensis* on *Microlaena stipoides* and *C. sporobolicola* on *Sporobolus australasicus*. Two species were described from other hosts, *C. coatesiae* from *Litchi chinensis* (Sapindaceae) and *C. colbranii* from *Crinum zeylanicum* (Amaryllidaceae). One species, *C. reesii*, was described from an isolate obtained from an air sample. Furthermore, DNA sequences derived from ex-type cultures have supported the generic placement of *C. neoindica* and the transfer of *Drechslera boeremae* to *Curvularia*.

It is not known whether the species described here are pathogens, endophytes or saprobes. It is also unclear as to whether these species are native or introduced. *Curvularia beasleyi* and *C. beerburrumensis* were both isolated from a native Australian grass species, as well as an exotic host. Some grass species have been reported to be associated with multiple *Curvularia* species, such as *Chloris* and *Cynodon*, with nine and 15 species, respectively. Many of the published records on *Chloris* and *Cynodon* have not been verified by molecular analyses. The number of new species described from non-Australian grasses indicates a need for a molecular-based reassessment of previous host-species records. The description of these species provides a foundation upon which additional sampling and accumulation of molecular data will improve knowledge of the host ranges and ecological roles of helminthosporioid fungi in Australia and overseas.

## Supplementary Material

XML Treatment for Curvularia beasleyi

XML Treatment for Curvularia beerburrumensis

XML Treatment for Curvularia boeremae

XML Treatment for Curvularia coatesiae

XML Treatment for Curvularia colbranii

XML Treatment for Curvularia eragrosticola

XML Treatment for Curvularia kenpeggii

XML Treatment for Curvularia lamingtonensis

XML Treatment for Curvularia mebaldsii

XML Treatment for Curvularia petersonii

XML Treatment for Curvularia platzii

XML Treatment for Curvularia reesii

XML Treatment for Curvularia sporobolicola

XML Treatment for Curvularia warraberensis
